# Non-inferiority, randomised, open-label clinical trial on the effectiveness of transurethral microwave thermotherapy compared to prostatic artery embolisation in reducing severe lower urinary tract symptoms in men with benign prostatic hyperplasia: study protocol for the TUMT-PAE-1 trial

**DOI:** 10.1186/s13063-024-08409-x

**Published:** 2024-09-02

**Authors:** Anna Kristensen-Alvarez, Mikkel Fode, Hein Vincent Stroomberg, Kurt Krøyer Nielsen, Albert Arch, Lars Birger Lönn, Mikkel Taudorf, Steven John Widecrantz, Andreas Røder

**Affiliations:** 1 Department of Urology, Copenhagen University Hospital - Rigshospitalet, Copenhagen, Denmark; 2 Department of Clinical Medicine, University of Copenhagen, Copenhagen, Denmark; 3 Department of Urology, Copenhagen University Hospital - Herlev and Gentofte, Herlev, Denmark; 4 Department of Public Health, University of Copenhagen, Copenhagen, Denmark; 5 Department of Radiology, Copenhagen University Hospital - Rigshospitalet, Copenhagen, Denmark; 6 Department of Urology, Zealand University Hospital, Roskilde, Denmark

**Keywords:** Benign prostatic hyperplasia, Lower urinary tract symptoms, International Prostate Symptom Score, Patient-reported outcome measures, Prostatic artery embolisation, Transurethral microwave thermotherapy, Minimally invasive surgical therapy, Erectile function, Non-inferiority, Multicentre

## Abstract

**Background:**

One-fourth of men older than 70 years have lower urinary tract symptoms (LUTS) that impair their quality of life. Transurethral resection of the prostate (TURP) is considered the gold standard for surgical treatment of LUTS caused by benign prostatic hyperplasia (BPH) that cannot be managed conservatively or pharmacologically. However, TURP is only an option for patients fit for surgery and can result in complications. Transurethral microwave thermotherapy (TUMT) and prostatic artery embolisation (PAE) are alternative minimally invasive surgical therapies (MISTs) performed in an outpatient setting. Both treatments have shown to reduce LUTS with a similar post-procedure outcome in mean International Prostate Symptom Score (IPSS). It is however still unknown if TUMT and PAE perform equally well as they have never been directly compared in a randomised clinical trial. The objective of this clinical trial is to assess if PAE is non-inferior to TUMT in reducing LUTS secondary to BPH.

**Methods:**

This study is designed as a multicentre, non-inferiority, open-label randomised clinical trial. Patients will be randomised with a 1:1 allocation ratio between treatments. The primary outcome is the IPSS of the two arms after 6 months. The primary outcome will be evaluated using a 95% confidence interval against the predefined non-inferiority margin of + 3 points in IPSS. Secondary objectives include the comparison of patient-reported and functional outcomes at short- and long-term follow-up. We will follow the patients for 5 years to track long-term effect. Assuming a difference in mean IPSS after treatment of 1 point with an SD of 5 and a non-inferiority margin set at the threshold for a clinically non-meaningful difference of + 3 points, the calculated sample size was 100 patients per arm. To compensate for 10% dropout, the study will include 223 patients.

**Discussion:**

In this first randomised clinical trial to compare two MISTs, we expect non-inferiority of PAE to TUMT. The most prominent problems with MIST BPH treatments are the unknown long-term effect and the lack of proper selection of candidates for a specific procedure. With analysis of the secondary outcomes, we aspire to contribute to a better understanding of durability and provide knowledge to guide treatment decisions.

**Trial registration:**

ClinicalTrials.gov NCT05686525. Registered on January 17, 2023, https://clinicaltrials.gov/study/NCT05686525.

## Administrative information

Note: the numbers in curly brackets in this protocol refer to SPIRIT checklist item numbers. The order of the items has been modified to group similar items (see http://www.equator-network.org/reporting-guidelines/spirit-2013-statement-defining-standard-protocol-items-for-clinical-trials/).

**Table:** 

Title {1}	Non-inferiority, Randomised, Open-label Clinical Trial on the Effectiveness of Transurethral Microwave Thermotherapy Compared to Prostatic Artery Embolisation in Reducing Severe Lower Urinary Tract Symptoms in Men with Benign Prostatic Hyperplasia: Study Protocol for the TUMT-PAE-1 Trial
Trial registration {2a and 2b}.	NCT05686525 [ClinicalTrials.gov] [registered after the start of inclusion; 17 January 2023]
Protocol version {3}	Version 3.0 of 12/12/2022
Funding {4}	The study was initiated by the Urological Research Unit at Rigshospitalet and the Department of Urology at Herlev-Gentofte Hospital. The trial is funded by the Alfred Benzon Foundation. The investigators have no financial interests in the Foundation. All other expenses are financed by the participating departments.
Author details {5a}	^1^Dept of Urology, Urological Research Unit, Copenhagen University Hospital – Rigshospitalet, Copenhagen, Denmark; Dept of Clinical Medicine, University of Copenhagen, Copenhagen, Denmark ^2^Dept of Urology, Copenhagen University Hospital - Herlev and Gentofte, Herlev, Denmark; Dept of Clinical Medicine, University of Copenhagen, Copenhagen, Denmark ^3^ Dept of Urology, Urological Research Unit, Copenhagen University Hospital – Rigshospitalet, Copenhagen, Denmark; Dept of Public Health, University of Copenhagen, Copenhagen, Denmark ^4^Dept of Radiology, Copenhagen University Hospital – Rigshospitalet, Copenhagen, Denmark; Dept of Clinical Medicine, University of Copenhagen, Copenhagen, Denmark ^5^Dept of Urology, Zealand University Hospital, Roskilde and Næstved, Denmark A. Kristensen-Alvarez^1^ M. Fode^2^ H.V. Stroomberg^3^ Kurt Krøyer Nielsen^2^ Lars Lönn^4^ Mikkel Taudorf^4^ Steven John Widecrantz^5^ A. Røder^1^
Name and contact information for the trial sponsor {5b}	Andreas Røder andreas.roeder@regionh.dk
Role of sponsor {5c}	The funding source had no role in the design of this study and will not have any role during its execution, analyses, interpretation of the data, or decision to submit results.

## Introduction

### Background and rationale {6a}

Benign prostatic hyperplasia (BPH) is a noncancerous enlargement of the prostate gland and a frequent cause of bladder outlet obstruction (BOO) and lower urinary tract symptoms (LUTS) in ageing men. One-fourth of men older than 70 years have moderate to severe LUTS that impair their quality of life (QoL) [1]. The severity of LUTS is measured using quantitative symptom indices, with the International Prostate Symptom Score (IPSS) being the most widely used [2].

The management of LUTS caused by BPH is in many cases initially watchful waiting and non-pharmacological interventions [3]. If the result of conservative management is not satisfactory, pharmacological treatment with alpha-blockers alone or in combination with 5-alpha reductase inhibitors is recommended [3, 4]. Compared to alpha-blocker monotherapy, combined therapy has been shown to reduce the relative risk of BPH clinical progression in men with moderate to severe LUTS [4]. A subset of patients with persisting LUTS will need surgical therapy; transurethral resection of the prostate (TURP) is still regarded as the gold standard [3, 5]. However, TURP requires a spinal epidural or general anaesthesia and can result in complications and adverse events (AEs) such as blood loss, urethral strictures, incontinence, and retrograde ejaculation [6]. Consequently, several less invasive procedures have been developed for men who are not candidates for or do not wish to undergo TURP.

Transurethral microwave thermotherapy (TUMT) and prostatic artery embolisation (PAE) are minimally invasive surgical therapies (MISTs) performed in an outpatient setting without the need for general anaesthesia. Both treatments have proven effective in reducing severe LUTS due to BPH with approximately 10 points on the IPSS after 3 months [7–12]. It is however difficult to compare the effect of the two procedures based on previous research as the selection of candidates for the two treatments varies between studies. TUMT is an ablative technique where a specially designed urinary catheter with a microwave heat antenna is inserted in the urethra and used to destroy hyperplastic prostatic tissue [13]. TUMT has been shown to be an effective alternative to alpha-blockers for treating symptomatic BPH [14]. A study looking at long-term follow-up after TUMT showed that 10% needed additional treatment after 5 years [15]. In PAE, an interventional radiologist embolises the prostate arteries under fluoroscopy guidance [16], causing ischemia of prostatic tissue and consequently shrinkage of the gland. A recent study has shown that PAE provides better urinary and sexual score benefits than combined therapy with a prostate-selective alpha-blocker and 5-alpha reductase inhibitor, in patients with bothersome LUTS refractory to prostate-selective alpha-blocker treatment alone [17]. A study looking at long-term results after PAE has shown that 5 years after treatment 23.6% needed additional LUTS treatment [18].

To date, PAE has not been compared to TUMT in a randomised clinical trial [19]. In the TUMT-PAE-1 trial, the primary objective is to assess the effectiveness of the reduction in the IPSS of PAE compared to TUMT for the treatment of BPH. A challenge with MIST BPH treatment is the lack of guidance when selecting candidates for each procedure [10]. The secondary outcomes after short- and long-term follow-up, including side effects, functional outcomes, and QoL, are selected to provide information that could guide treatment options in the future.

## Objectives {7}

The primary objective of this trial is to investigate the non-inferiority of PAE to TUMT in reducing LUTS 6 months post-procedure. The secondary objective is to compare LUTS (IPSS), patient satisfaction with the treatment, QoL (International Prostate Symptom Score—Quality of Life (IPSS-QoL), EQ-5D-5L and BPH Impact Index (BII)), health economics (EQ-5D-5L), erectile function (International Index of Erectile Function—Erectile Function (IIEF-EF)), ejaculatory function (Danish Prostatic Symptom Score—sexual questions (DAN-PSS-1-sex)), incontinence (International Consultation on Incontinence Questionnaire—Urinary Incontinence Short Form (ICIQ-UI-SF)), uroflowmetry outcomes, post-procedure duration of catheterisation, prostate volume (PV), prostate-specific antigen (PSA) and creatinine, incidence of side effects, de novo use of pharmacological LUTS treatment, and surgical re-treatment rates of TUMT and PAE during the 5 years following the procedures. The objective of this comparison is to create a reference for clinicians in patient counselling when choosing a MIST for the treatment of BPH.

## Trial design {8}

This study is designed as a pragmatic multicentre, non-inferiority, open-label randomised clinical trial comparing the effectiveness of TUMT versus PAE in reducing the symptoms of BPH. A non-inferiority design has been chosen as the treatments are expected to perform similarly in effectiveness. If non-inferiority is demonstrated, the analysis of our secondary outcomes will be able to support decision-making for the individual patients needs when choosing a MIST for the treatment of BPH. PAE will be tested for inferiority with TUMT as the reference. PAE is expected to be inferior to TUMT by + 1 point on the IPSS within the non-inferior margin allowing the favour of TUMT of + 3 points in the IPSS. Patients will be randomised with a 1:1 allocation ratio between the two treatments.

## Methods: participants, interventions, and outcomes

### Study setting {9}

The TUMT-PAE-1 trial will be performed at academic hospitals in northern Europe. Eight centres are planned to be included in the trial.

From the Capital Region and Region Zealand of Denmark, the included hospitals are Rigshospitalet, Herlev and Gentofte University Hospital, and Zealand University Hospital. From the Region of Southern Denmark, we expect to include Odense University Hospital, and from the Central Denmark Region, Aarhus University Hospital. We hope to also include Helsingborg Hospital and Karolinska University Hospital in Sweden and Oslo University Hospital in Norway.

### Eligibility criteria {10}

We have kept the eligibility criteria to a minimum for a pragmatic framework to reflect real-life patient populations.

#### Inclusion criteria

- Men ≥ 40 years.
- Ability to understand and the willingness to sign an informed consent.
- Diagnosis of LUTS secondary to BPH refractory to/contraindicated for medical treatment or patient preference for TUMT/PAE.
- Severe urinary symptoms on IPSS (IPSS ≥ 20), an indwelling catheter, or intermittent catheterisation. In the last two cases, the baseline IPSS is set to 35 points.
- A maximum flow rate (Qmax) ≤ 15 mL/s.
- Prostate volume at minimum 50 mL measured by transrectal ultrasound measurement (TRUS) or MRI.

#### Exclusion criteria

- Active bladder cancer with any other grading than pTa low-grade.
- Prostate cancer on any other treatment plan than active surveillance or watchful waiting.
- Previous pelvic radiation.
- Bladder stones (inclusion is allowed after removal).
- Current urethral stricture or bladder neck contracture.
- Neurogenic LUTS.
- Documented bacterial prostatitis in the last year.
- Severe atherosclerotic vessels or other pathology preventing catheter-based intervention (as rated on computerised tomography angiography (CTA) by an interventional radiologist).
- Allergy to iodinated contrast media.
- Renal failure, defined as eGFR < 35 mL/min.
- High bleeding risk, defined as spontaneous INR > 1.6.
- Contraindication to conscious sedation (if requested by the patient).
- Large prostate median lobe as defined by the treatment physician.
- Urethral colliculus to bladder neck length < 35 mm.

### Who will take informed consent? {26a}

Patients referred with LUTS due to BPH with urinary obstruction planned to be treated with a MIST will be evaluated for eligibility to participate in the study by the treatment-responsible physicians at the participating departments of urology. After the assessment of the patient’s eligibility, relevant study material will be provided to the patient. If the patient gives oral consent, the treatment-responsible physician will pass on the necessary personal information to the principal investigator (PI) at the respective centre. The patient will be invited for a screening visit with the research physician to discuss any questions and sign the informed consent. The patient will be offered a minimum of 24 h of decision time. If the patient wishes a prolonged decision period, a new appointment will be arranged.

### Additional consent provisions for collection and use of participant data and biological specimens {26b}

N/a. No further consent is needed since no biological material will be stored after analysis.

## Interventions

### Explanation for the choice of comparators {6b}

No standard of care exists for the included patient group. Current research indicates that TUMT and PAE are two MISTs with similar results in the efficacy of reducing LUTS. Thus, the clinical decision about which treatment is offered to the patient is settled at each treatment centre. Alternative treatments, which may be offered to the TUMT or PAE patient group, include transurethral water vapour thermal therapy (Rezūm), clean intermittent catheterisation, or surgery with general anaesthesia/spinal epidural including TURP or open/robot-assisted prostatectomy (a.m. FREYER).

### Intervention description {11a}

The patients will be managed according to operating procedures for TUMT and PAE at each centre. Both procedures are conducted under local anaesthesia in the outpatient department. Usually, the patient can be discharged from the hospital on the same day. If the patient cannot be discharged on the same day, he will be admitted to the urological ward. Below is a general description of the two procedures.

#### Arm 1: TUMT

TUMT is performed by a urologist and/or a trained urological nurse. In TUMT, a specially designed instrument that sends out microwave energy is inserted inside the prostate through the urethra. Cooling fluid circulates the instrument to prevent heat from damaging the wall of the urethra. To prevent the temperature from getting too high outside the prostate, a temperature sensor is positioned around the penis and inside the rectum. If the temperature reaches the safety limit, the microwave generator’s output will shut off automatically. Utilising the ProstaLund CoreTherm Device (ProstaLund, Lund, Sweden) microwave delivered by ProstaLund Feedback Treatment is used to heat the prostate at a range of 50–60 °C, destroying hyperplastic prostate tissue. As the prostate heals, it will shrink, reducing the blockage of urine flow and the symptoms of BHP. After the treatment, a transurethral catheter is inserted. This will be removed at the physician’s discretion when spontaneous voiding is achieved with an acceptable residual volume (generally < 150 mL), typically within 4 weeks.

#### Arm 2: PAE

In PAE, an interventional radiologist will insert a small catheter into the vessels that supply blood to the prostate. Habitually arterial access is reached by a unilateral femoral sheath placed in the right common femoral artery under local anaesthesia. Digital subtraction angiography of the pelvic arteries is performed to assess the origin points of the internal iliac arteries and to map the vessels feeding the prostate. Pelvic arterial anatomy is complex and prostatic arteries exhibit a noticeable variation of origin, are often duplicated, and provide collaterals to the opposite half of the prostate and nearby structures. In addition, they are often markedly tortuous, making catheter advancement difficult. Cone beam computed tomography can be applied to assure correct placement or prevent non-target embolisation. Tiny embolisation particles are injected through the catheter and into the blood vessels to reduce the blood supply to the prostate. This procedure is intended at both sides of the prostate. Following the procedure, the prostate will shrink reducing the symptoms of BPH. A transurethral catheter is inserted before the procedure. In patients without a permanent catheter before PAE, this will be removed after the procedure. Patients with a permanent catheter before PAE will keep the catheter until spontaneous voiding is achieved with an acceptable residual volume (generally < 150 mL), typically within 4 weeks.

### Criteria for discontinuing or modifying allocated interventions {11b}

The patient can withdraw the informed consent and leave the study at any time without explanation. If the patient withdraws consent, no further data will be collected. The data already collected until that point will be stored and used for study purposes. If the patient requests to change the treatment option after randomisation, he will be referred to his respective department of urology for his preferred treatment option and included in the analysis as intention-to-treat. The study can be closed prematurely if any untoward patient complication is observed in either treatment arm, defined as serious adverse events (SAEs) resulting in organ failure or death.

### Strategies to improve adherence to interventions {11c}

N/a. As the interventions are surgical and performed as one-time procedures, no strategies to improve adherence were formed.

### Relevant concomitant care permitted or prohibited during the trial {11d}

As a pragmatic study, any concomitant care that the treating physician finds relevant is permitted during the trial but will be registered.

### Provisions for post-trial care {30}

The patients will be followed for 5 years at the urological research unit at the respective centre; any need for ancillary or post-trial care will be looked after.

### Outcomes {12}

The outcome measures used in this trial generally follow the registry network for surgical treatment of BPH recently developed [20]. The primary outcome is the mean IPSS of the TUMT and PAE arm 6 months after the procedure. The IPSS is a validated, reproducible, patient-reported outcome measure (PROM) to assess LUTS severity and response to treatment. This measure was selected as the primary outcome as it is the most clinically relevant and internationally accepted PROM for assessing LUTS [21].

The secondary outcomes include the following:

- The mean IPSS of the two arms at all other follow-up occasions than the primary outcome.
- The mean procedure time for the conduction of TUMT and PAE as well as the calculated necrosis.
- Health-related quality of life measured by the mean IPSS-QoL, BII, and EQ-5D-5L. IPSS-QoL assesses the quality of life associated with symptoms from urinary problems. The BII is a self-reported, validated, disease-specific instrument for quantifying the interference of LUTS on the patient’s mental health and activity. As a measure of QoL, it is used to determine treatment outcomes in men with symptomatic benign prostatic hyperplasia [22]. The EQ-5D is the most widely applied instrument for health-economic assessment worldwide. Its index values are used in the estimation of quality-adjusted life year gains in economic evaluations of healthcare interventions [23].
- Mean satisfaction with treatment by a five-level bipolar Likert scale.
- Erectile function measured by the mean IIEF-EF score. The IIEF-EF domain score is a PROM used to measure erectile performance and assess disease severity in efficacy trials. It distinguishes between men with and without erectile dysfunction (ED), classifies levels of ED severity, and has the requisites to detect treatment-related changes in patients with ED [24, 25].
- Ejaculatory function measured by the mean DAN-PSS-1-sex. The DAN-PSS-1-sex is a validated instrument to determine erectile and ejaculatory function and its impact on the well-being of the patient [26].
- Incontinence measured by the mean ICIQ-UI-SF. The ICIQ-UI-SF is validated for evaluation of the frequency, severity, and impact on QoL of urinary incontinence [27].
- Mean prostate volume (PV) measured by TRUS in mL.
- Mean uroflowmetry outcomes of the two arms. We will be looking at voided volume, Qmax, and residual urine. Uroflowmetry is a non-invasive procedure used to measure the flow of urine throughout micturition. It provides useful information about the total voiding function and is used for diagnosis of lower urinary tract dysfunctions and evaluation of treatment response. Qmax is the value of the highest flow rate measured during the test; it will be set to zero if the patient has an indwelling catheter. Transabdominal ultrasound will be used to assess post-voided residual urine as it is accurate and non-invasive [28].
- Post-procedure catheterisation events, including the placement of an indwelling urinary catheter and the use of intermittent catheterisation. Catheterisation will be expressed as the reason for placement, duration, and frequency (in case of intermittent catheterisation). If an indwelling catheter is placed after the procedure, the post-procedure duration of catheterisation will be defined as days from the procedure till indwelling catheter cessation. Catheterisation for any other reason during the follow-up period will also be evaluated.
- Prostate-specific antigen (PSA) and creatinine. The absolute value of serum PSA (ng/mL) has a good predictive value for the determination of prostate volume. Continued PSA measurements assist in the detection of any de novo prostate cancer or progression of patients in active surveillance or watchful waiting. A rise in serum creatinine can be a result of BPH with urinary retention. Serum creatinine aids in the identification of any affection of the kidney from the procedures.
- De novo use of pharmacological LUTS treatment and surgical re-treatment rates will be collected from the patient’s health record.
- Incidence at 3 months post-procedure of urinary tract infection, acute urinary retention, dysuria, haematuria, post-embolisation syndrome (PES), hospital admission, and hospitalisation time. PES is defined as occurring within 48 h after surgery and consisting of influenza-like symptoms, pelvic pain, nausea, dysuria, or transient worsening of LUTS. Data on AEs and side effects will be collected from the patient’s health record and the follow-up visit at 3 months. Surgical complications will be categorised following the Clavien-Dindo grading [29].

In Table 1, the specific time points for collection of secondary outcomes can be appreciated.

**Table 1:** Time schedule

Visit	Screening	Intervention	FU 1	FU 2	FU 3	FU 4	FU 5	FU 6
Day	− 365 to − 2	0	1 month (± 5 days)	3 months (± 14 days)	6 months (± 14 days)	1 year (± 14 days)	2 years (± 14 days)	5 years (± 14 days)
**Screening activities**								
Informed consent	**X**							
Inclusion/exclusion	**X**							
Randomisation	**X**							
Demographics	**X**							
Medical history	**X**							
**Procedures**								
CTA	**X**							
TRUS/MRI (prostate volume)	**X**			**X**	**X**	**X**	**X**	
Blood test (PSA + creatinine)	**X**			**X**	**X**	**X**	**X**	
Uroflowmetry (flow + residual urine)	**X**			**X**	**X**	**X**	**X**	
**Assessments**								
Likert scale			**X**	**X**				
PROMs (IPSS, IPSS-QoL, BII, EQ-5D-5L, ICIQ-UI-SF, IIEF-EF, DAN-PSS-1-sex)	**X**			**X**	**X**	**X**	**X**	**X**
Catheter use	**X**			**X**	**X**	**X**	**X**	
Technical details of the procedure				**X**				
AEs/SAEs				**X**				
Re-treatment rates						**X**	**X**	**X**

*CTA* computed tomography angiography, *TRUS* transrectal ultrasound, *MRI* magnetic resonance imaging, *PSA* prostate-specific antigen, *PROM* patient-reported outcome measure, *IPSS* International Prostate Symptom Score, *IPSS-QoL* International Prostate Symptom Score—Quality of Life, *BII* Benign Prostatic Hyperplasia Impact Index, *ICIQ-UI-SF* International Consultation on Incontinence Questionnaire—Urinary Incontinence Short Form, *IIEF-EF* International Index of Erectile Function—Erectile Function, *DAN-PSS-1-sex* Danish Prostatic Symptom Score—sexual questions, *AE* adverse event, *SAE* serious adverse event

### Participant timeline {13}

#### Screening and inclusion

At the screening visit, the research physician will determine whether the patient is eligible to be included in the study by assessing inclusion and exclusion criteria. At inclusion, baseline data will be obtained following Table 1.

##### Follow-up management

Patients will be followed for 5 years post-procedure, with a total of four follow-up visits to the outpatient clinic at 3, 6, 12, and 24 months. The results from the long-term follow-up at 5 years will be completed with digital PROMs and assessment of the patient’s health record. Outcome data will be collected in accordance with Table 1. To complete the procedures and PROMs for the follow-ups at 3 months to 5 years, a 28-day window will be allowed, defined as 14 days before and 14 days after the due date. For the 1-month follow-up, the permitted window will be 10 days, 5 days before and 5 days after the due date.

### Sample size {14}

A non-inferiority study design was chosen based on several case studies on the efficacy of TUMT and PAE in men with similar inclusion criteria as this trial. From a review of TUMT, a study with a baseline IPSS above 19 and follow-up after 3 months was selected [30]. In this study, the IPSS 12 weeks after TUMT had decreased to 11.3 with a standard deviation (SD) of 6.3 [8]. With PAE, a recent independent study has demonstrated an IPSS at baseline of 19.4 with an SD of 6.3. Twelve weeks after the procedure, the IPSS declined to 10 with an SD of 6.5 [7]. The difference of approximately 1 IPSS point between the two treatments holds for 6- and 12-month follow-up according to the data available in systematic reviews [10, 12, 31]. Due to resource constraints of our trial and the great variation in SD across TUMT and PAE trials, we have chosen an SD of 5 [30, 32]. The sample size calculation was based on “Sample sizes for clinical trials with normal data. *Statist. Med.* 2004; 23:1921–1986” by Julious [33]. The size was calculated using the package “PowerTOST” in R for the sampleN.noninf() function in R set to parallel design. We used a one-sided significance level of 0.025. Power was set at 80%. Assuming a difference in mean IPSS after treatment of + 1 point in the favour of TUMT with an SD of 5 and a non-inferiority margin set at the border for a clinically non-meaningful difference of + 3 points in the favour of TUMT [34, 35], the calculated size was 100 per group, giving a total size of 200 patients. Dropouts are defined as patients who after inclusion are lost to follow-up for the primary outcome. To compensate for an anticipated dropout rate of 10%, the study will include 223 patients.

### Recruitment {15}

The enrolment period is expected to be 3 years. The study will be terminated after the last follow-up of the last patient. The total study duration is expected to be 8 years. Patients will be recruited from the urological outpatient clinics of the participating centres. The treatment-responsible physician will alert the patient when he is selected for MIST BPH treatment. The number of patients treated with either TUMT or PAE in the Capital Region of Denmark every year exceeds the target sample size. As it is a pragmatic trial, most of these patients will be eligible for inclusion. As the enrolment period is 3 years, we expect the flow of patients in the clinic to be sufficient to cover the sample size. By having multiple centres participate in the study, we prevent bias and ensure sufficient inclusion.

## Assignment of interventions: allocation

### Sequence generation {16a}

The study will randomise patients in a 1:1 manner to either Arm 1: TUMT or Arm 2: PAE following a computer-generated randomisation schedule. Randomisation will be performed as block randomisation including blocks of varying sizes with site stratification using the randomisation module in REDCap (Research Electronic Data Capture). Details on block sizes will not be disclosed to ensure the concealment for those enrolling patients.

### Concealment mechanism {16b}

REDCap allocates the patient when baseline measures have been completed and the informed consent signed, in that way the allocation concealment will be guaranteed.

### Implementation {16c}

In this study, we will implement complete separation of the individuals involved in sequence generation and assignment of treatment. The randomisation sequence is generated by the trial statistician who will not be involved in enrolling patients. In this way, randomisation will be conducted without the influence of the PI or trial physicians. The block size will be concealed until the primary endpoint will be analysed. The list will be uploaded to REDCap without the possibility that those enrolling and assigning participants will obtain access to the list.

## Assignment of interventions: blinding

### Who will be blinded {17a}

N/a. This is an open-label trial; both study personnel and patients will be aware of the received treatment. Due to the physical component of the surgical procedures and outcome assessments, blinding of the patients, surgeons, and outcome assessors has not been possible in our setting. The data analyst will be blinded by having an independent statistician who will be blinded to treatment group allocations conducting the analysis.

### Procedure for unblinding if needed {17b}

N/a. This is an open-label trial; both study personnel and patients will be aware of received treatment.

## Data collection and management

### Plans for assessment and collection of outcomes {18a}

**Fig. 1:**
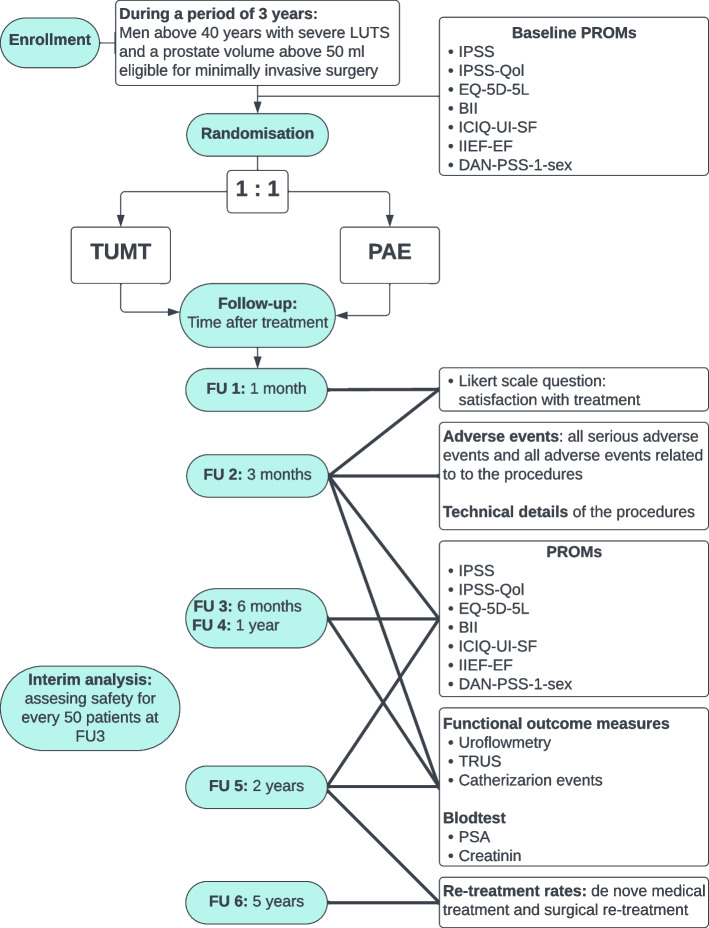
Trial profile

Data from assessments and procedures will be collected following Table 1 and Fig. 1. All trial data will be collected in REDCap, a secure web application for building and managing databases and online surveys. Project staff will be trained in completing the REDCap database by the PI and all study groups will follow a REDCap manual for data collection. Predefined definitions for complications and AEs will be specified in the REDCap database to get homogeneous responses for AE outcomes. PROMs will as a first option be distributed to the patients by a REDCap link sent to their phone as an SMS via a secure third-party service. All questionnaires will be included in the same survey string via the auto-continue function in REDCap; this ensures data collection from all PROMs. Data from the questionnaires will be collected in REDCap directly. PROMs and data collection instruments are chosen to be reliable, valid, and have inter-rater reliability with previous randomised clinical trials in BPH research. Most of the instruments used here are included in the registry network for surgical treatment of BPH [20].

### Plans to promote participant retention and complete follow-up {18b}

For an improved adherence to follow-up protocol, study benefits and importance will be emphasised at inclusion. A well-functioning, organised, and persistent research team with trained research nurses will have the responsibility of data registration. Surveys are preferably distributed digitally as it has shown a greater adherence [36]. However, the data collection methods can be tailored to the participant’s needs. PROMs can be distributed by text message, e-mail, or paper format and inclusion visits can be facilitated in person and/or by phone. REDCap Automated Survey Distribution Tool will be used to ensure distribution at the right time. This will also send out reminders after 3 days if a survey has not been completed. If questionnaires are still not being filled out, to ensure the primary outcome is completed, the patient will complete the surveys at a visit to the outpatient clinic or will be contacted by the study personnel. Reminders will not be sent to those participants who have completed their survey, opted out, or have been marked as not active in the study.

### Data management {19}

Data collection and entry are combined as electronic data capture is used in the TUMT-PAE-1 trial. Various REDCap tools will be used to ensure the accuracy of data entry and coding. Field Notes will be used to define the metric unit accepted and give short definitions of the requested variable. Field Annotations provide a thorough explanation of each variable and can be used for reference purposes regarding the denotation of the field and how data should be entered and assessed. Field Annotations can be found in the electronic Data Dictionary Codebook made available for study personnel through REDCap. Dropdown lists, radio buttons, and checkboxes will be preferred for data entry to ease data storage, review, and analysis. When text boxes are used, REDCap Validation Rules will be applied, and an error will be displayed if the entry does not meet the criteria of the data type and value. This will promote data quality by narrowing down the input options of text box fields. The Required Field function is used so that data entry cannot be marked as complete before all data is captured; this will ensure collection of all outcome measures. REDCap users will only gain access to the project when assigned by the PI, who will also define the roles of each user with limited privileges. All data will be stored in REDCap for 10 years after the trial ends. No data from this trial will be sent abroad.

### Confidentiality {27}

Before informed consent, the subject’s health records will be screened for inclusion only by the treatment-responsible urologist. Relevant data collected before the informed consent will be shared with the study team. Signing the informed consent gives the study researchers direct access to the subject’s health records to retrieve information necessary to conduct the study. By Danish law, all data will be made available to third parties for control purposes (external monitoring, quality control, etc.). Personal data will be stored electronically in REDCap following the Danish Data Protection Regulation (Databeskyttelseforordningen), the Danish Data Protection Act (Databeskyttelseloven), and the Danish Health Law (Sundhedsloven). Several tools in REDCap will be used to protect confidentiality before, during, and after the trial. In this multicentre study, data access groups (DAGs) will be created in REDCap for restriction of viewing. DAGs will preserve data confidentiality by ensuring that study personnel can only view data from their centre. To protect data and maintain its validity, data access and privileges will be limited by defining user rights and roles in the REDCap database. User rights will be restricted according to the minimum necessary for individuals to complete their assigned tasks. Only the PI will be able to view all records and extract data. Personal data according to the General Data Protection Regulation (GDPR) will be pseudonymised with a serial code ID number. If the patient withdraws consent, no further data will be collected. Data collected up until withdrawal will be stored and used for study purposes. Patients possess the right to be informed about the location of their data storage, the entities utilising it, and the specific purposes for which it is employed. At any given time, they retain the prerogative to request a copy of their study data, either for personal use or for sharing with others. Furthermore, patients are entitled to rectify any inaccuracies present in their personal data. They also have the authority to object to or restrict the utilisation of their data and can demand its deletion as needed.

### Plans for collection, laboratory evaluation, and storage of biological specimens for genetic or molecular analysis in this trial/future use {33}

N/a. No biological material will be stored after analysis.

## Statistical methods

### Statistical methods for primary and secondary outcomes {20a}

The primary endpoint will be evaluated using a 95% confidence interval against the predefined non-inferiority threshold of + 3 points in the IPSS. PAE is expected to be inferior to TUMT by + 1 point on the IPSS within the non-inferior margin allowing the favour of TUMT of + 3 points in the IPSS. To control for initial differences between groups that might affect the result, the primary analysis will be adjusted for site and baseline IPSS. Normal distribution is predicted, and a parametric test will be used. The difference in mean IPSS is expected to be tested with analysis of covariance (ANCOVA) for non-inferiority. Secondary outcomes will be represented descriptively and analysed according to the data type (categorical or continuous) and their distribution. Likely data representations are median with interquartile range and mean with SD for continuous variables and proportions of categorical variables. We plan to compare the secondary outcomes statistically using Student’s *t*-test, chi-square test, and linear mixed models. Statistical analysis will be undertaken using R version 3.2 or later if available. The Eq. 5d package in R will be used to perform the calculation for the economic evaluation of health care from the EQ-5D-5L. In case of deviations from the statistical analysis plan, the information on clinicaltrials.gov will be updated.

### Interim analyses {21b}

The trial will be conducted as a traditional fixed-sample design. An interim analysis of the safety of both procedures will be performed for every 50 cases at 6-month follow-up. The trial-initiating group will have access to the interim results and make the final decision if the trial is to be terminated.

### Methods for additional analyses (e.g. subgroup analyses) {20b}

To address disparities between study groups, we plan to perform additional adjusted analyses correcting for baseline prostate size, age, and Qmax. No additional subgroup analysis is planned at the initiation of this trial.

### Methods in analysis to handle protocol non-adherence and any statistical methods to handle missing data {20c}

The study is analysed as intention-to-treat. As data is expected to be missing completely at random, missing data will be handled by pairwise deletion.

### Plans to give access to the full protocol, participant-level data, and statistical code {31c}

All study data including study protocol, statistical analysis plan, informed consent form, and clinical study report can be shared when a proper agreement is formed according to the European Union GDPR protection statement.

## Oversight and monitoring

### Composition of the coordinating centre and trial steering committee {5d}

The project is coordinated and run day to day in collaboration with each urological research unit, the Department of Urology, and the Department of Diagnostic Radiology at the respective centres. PIs at each centre will monitor correct data collection following the ICH GCP guidelines.

### Composition of the data monitoring committee, its role and reporting structure {21a}

The data monitoring is done by the research team at the Urological Research Unit at Rigshospitalet and Herlev and Gentofte University Hospital. As part of the Urological Research Unit at Rigshospitalet and Herlev and Gentofte University Hospital, the coordinating investigator (CI) will periodically review the accumulating data and, together with the research team, determine if a trial should be modified or discontinued. The CI will report to the ethical committee within 90 days of study termination or completion. As the risk profile of this trial is predicted to be minimal, a formal data monitoring committee has not been established.

### Adverse event reporting and harms {22}

#### Adverse events definition

##### AEs

An AE is any untoward medical occurrence in a patient or to whom a medical product or surgical procedure has been administered, including occurrences that are not necessarily caused by or related to the procedure. The study compares two acknowledged treatments with a known spectrum of side effects. Therefore, the assessment of AEs is not based on spontaneous reporting but on these specific expected AEs occurring from the procedure until the first follow-up (0–3 months). The most common side effects of TUMT include urinary tract infection (0–30% [37]), urinary retention (0–12% [38]), dysuria (0–6.7% [14]), post-procedure haematuria (0–26.8% [14]), haematuria requiring additional treatment (0–1.24% [14]), retrograde ejaculation (0–22.2% [12]), and ED (0–9% [37]). Compared to TURP, men undergoing TUMT are significantly less likely to require blood transfusion and experience retrograde ejaculation [12]. The most common side effects of PAE include urinary tract infection (0–13.8% [39]), late acute urinary retention (0–10% [40]), PES (0–100% [39]), access-site hematoma (2–12% [41]), pseudoaneurysm (0.5 to 6.3% [42]), and ED (0–2% [7]). Major complications following PAE such as non-target embolisation and vessel injury with subsequent bleeding are rare with an overall incidence of 0.5% [43]. Surgical complications will be registered in REDCap by the study personnel and classified using the Clavien-Dindo classification [29].

##### SAEs

A SAE is an AE or adverse reaction that results in death, is life-threatening, requires hospitalisation or prolonged existing hospitalisation, or results in persistent or significant disability or incapacity. SAEs during the study (0–3 months from the procedure) are reported on a specific SAE Case Report form in REDCap. Patient register information from inpatient care will be used to assess the frequency, duration, and cause of hospitalisation. Each SAE will be classified by the research physician according to intensity as mild, moderate, or severe. It will be classified as mild when the SAE is acceptable, the subject is aware of symptoms or signs, but they are easily tolerated. As moderate when it is disturbing and causes discomfort enough to interfere with usual daily activity. And as severe when it is unacceptable, the subject is incapable of working or doing usual daily activities. SAEs will also be classified by their likelihood of a causal relation with the given per-protocol procedure. In an unlikely causality, the event is most likely related to an aetiology other than the treatment under study. In a possible causality, a causal relationship is conceivable and cannot be dismissed. In a probable causality, there is good reason and sufficient documentation to assume a causal relationship.

#### Adverse event reporting

The REDCap alerts tool will be used to notify the PI when an SAE has been reported in the database. The PI at each centre is responsible for reporting any unexpected SAEs to the ethics committee within 7 days. This report will be accompanied by remarks on any consequences of the trial. Throughout the total study duration, it is also the PIs responsibility to submit annual safety reports to the ethics committee of all serious expected and unexpected side effects and all serious events that have occurred during the period. This report will include an evaluation of the safety of the trial participants. The PI will keep study personnel informed of any safety issues that arise during the trial.

#### Radiation exposure

##### Fluoroscopy

Fluoroscopy is part of a standard protocol when performing PAE. Fluoroscopy relies on the use of X-rays for image formation. Radiation has inherent deterministic and stochastic effects. Deterministic effects are observed once a threshold dose is reached and may result in, i.e. hair loss or skin injury. So far, only one report on radiation-induced dermatitis after PAE and exposure to 8024 Gy∙cm^2^ has been published [44]. The stochastic effect is induced by chance with increasing dose which may cause radiation-induced cancer. Dose-area-product (DAP) is a common measure for the total exposure to the patient. The radiation doses in PAE are similar to those in other complex interventional procedures with a mean total DAP of around 180 Gy*cm^2^ (95% confidence interval 125–262 Gy*cm^2^), which is equal to a mean effective dose of 46 mSv (range 35–110 mSv) using median conversion factor of 0.26 mSv/Gy*cm^2^ as used in other abdominal embolisation procedures [45, 46].

##### Iodinated contrast media

An iodinated contrast media, iodixanol (Visipaque® 270 mg/mL), is used during embolisation. Iodixanol is a dimeric, isosmolar, nonionic, water-soluble, iodinated X-ray contrast agent for intravascular administration. The most common side effect is minimal discomfort at the injection site (< 30%). Side effects associated with iodixanol are usually mild to moderate and of transient character. Serious side effects and deaths are extremely rarely seen and may include acute-on-chronic renal failure, acute renal failure, anaphylactic or anaphylactoid shock, allergic acute coronary syndrome (Kounis syndrome), cardiac or cardiopulmonary arrest, and myocardial infarction. Cardiac responses may be enhanced by an underlying disease or the study itself. Fatal cases of lactic acidosis have been described when iodinated X-ray contrast agents are given to patients in metformin therapy with elevated serum creatinine; local guidelines will be followed in such cases. Hypersensitivity reactions may occur as respiratory or cutaneous symptoms such as respiratory distress, rash, erythema, hives, pruritus, severe or toxic skin reactions, angioedema, hypotension, fever, laryngeal oedema, bronchospasm, or pulmonary oedema. Cases of vasculitis and Stevens-Johnson-like syndrome have been seen in patients with autoimmune diseases. Serious hypersensitivity reactions and anaphylactoid reaction/shock are rare (0.1–1%) [47]. Side effects can occur either immediately after injection or up to a few days later. Hypersensitivity reactions may occur independently of dose and mode of administration, and mild symptoms may be the first signs of a severe anaphylactic reaction/shock. In such cases, administration of the contrast agent will be discontinued promptly. A minor transient increase in serum creatinine is common after iodinated contrast agents but is usually of no clinical relevance.

### Frequency and plans for auditing trial conduct {23}

The CI will perform periodic independent reviews of core trial processes and documents to confirm adherence to ICH GCP guidelines. The CI will explore the trial dataset and perform site visits to the participating centres. Audits might also be conducted by the Danish National Centre for Ethics.

### Plans for communicating important protocol amendments to relevant parties (e.g. trial participants, ethical committees) {25}

Important protocol modifications will be communicated promptly to the relevant parties, including the Danish National Centre for Ethics, and trial registries will be updated.

After the informed consent has been signed, patients will be informed about the trial’s effects, risks, side effects, complications, disadvantages, substantial design changes relative to the patient’s safety, eventual consequences for the patient (if practically possible and if the patient wishes it), and results. Important information about the patient’s health (unless the patient states clearly that he does not want to receive the information) and the reason for trial termination, if the trial is cancelled prematurely, will be shared with the patient.

## Dissemination plans {31a}

Trial results will be published in peer-reviewed international journals or otherwise made publicly available regardless of whether they show to be positive, negative, or non-significant. The trial results will be published open access. All published data will be anonymised and there will be no personal identification in the presentation of results.

To keep the scientific integrity of the project, the individual centres are not expected to report their data alone. Data from all centres will be analysed as a whole. An effort will be made to reduce the interval between the completion of data collection and the release of study results. The results of the long-term follow-up will be published as separate articles when they are available.

Publications are expected to protect the integrity of the major objectives of the study. Therefore, any non-powered data that might indicate one treatment is better than the other will be treated as such in order not to bias participants or healthcare. No publication restrictions/constraints were imposed by the funding body. The initiating research group at the Urological Research Unit at Rigshospitalet and Herlev and Gentofte University Hospital will determine the timing and place of presentation and publication of endpoint data. Each paper will be reviewed internally.

## Discussion

Surgical treatment is an essential part of LUTS management. In recent years, several MISTs have been developed for the treatment of BPH in men refractory to conservative and medical treatment, or in cases of absolute indication for surgery. These procedures seek to provide safe and effective surgical alternatives when TURP is not an option, or the patient does not wish to undergo TURP. With this trial, the effectiveness of reducing LUTS of TUMT and PAE will be compared. TUMT and PAE are MISTs where previous research indicates a similar reduction in LUTS. It has been shown that both TUMT and PAE are superior to sham procedures [8, 40]. Previous research comparing TUMT and PAE to the reference standard has shown an early reduction in symptoms comparable to TURP [7, 37]. However, the question of the durability of these procedures remains [10, 48].

An advantage of TUMT and PAE compared to resection or enucleation of the prostate is that these MISTs are performed in an outpatient setting with no need for spinal or general anaesthesia. As the global population ages, with a growing number of individuals reaching 65 years and older, there is a concurrent increase in the prevalence of comorbid conditions in this age group. In response to these demographic shifts, there is a rising demand for medical techniques that circumvent the necessity for general anaesthesia. A population that is ageing will need more LUTS treatment and in many cases preferably minimally invasive. Currently, the European Association of Urology guideline does not include any recommendations for surgical procedures without spinal or general anaesthesia for larger prostates (> 70 cc) [3]. It has been predicted that the stability of the results of IPSS reduction after PAE could be related to the prostate size, in the way that the results are more durable in larger prostates [17, 49]. Including only patients with prostates above 50 mL, we hope to avoid this bias and contribute to treatment guidelines for this patient group. As outpatient procedures, TUMT and PAE bring down the risk of immobilisation and infection in a hospital setting. These are important factors for the older demographic and a health system under pressure.

There is a decreasing trend in the use of TUMT which could affect the implication of this study. However, TUMT has been shown to have similar short-term effects as TURP and only the lack of knowledge about the long-term effect, has been found to decrease its use [50]. With our long-term follow-up, we hope to contribute to answering the question of the durability of TUMT as well as PAE. We believe that this comparison analysis has great relevance in the research of MIST options by assisting in the placement of these treatments in the landscape of BPH treatment. Based on their safety and efficacy profile, TUMT and PAE may fill a therapeutic niche in the management of LUTS between pharmacotherapy and more invasive surgical treatment. These MISTs could have a place for patients willing to undergo a MIST as an alternative to requiring life-long medication, even if beneficial results are short-term. A recent study proposes that MISTs might be used in the same line as combined pharmacological therapy and obtain better results in BOO symptoms and sexual function [17].

The main strengths of our study include the randomised treatment assignment that prevents selection bias and improves internal validity by ensuring equal distribution of confounding factors. Our pragmatic study design with a multicentre framework ensures external validity and minimises surgeon expert bias. Moreover, the interdisciplinary setting promotes a holistic understanding of the patient and the possibility to influence various fields. By choosing patient-centred trial outcomes, we ensure a comprehensive assessment of the benefits of the MISTs under investigation. A limitation of our study is that we only include patients with Qmax below 15 mL/s, risking the exclusion of patients with high-flow obstruction. However, only a minority of men with Qmax above 15 mL/s have BOO [28], making it unlikely that including these patients would change our results. We chose Qmax as an inclusion criterion as previous research recommends surgery based on Qmax below 15 mL/s [21]. We did not add urodynamics (multichannel cystometry) as a part of the inclusion criteria as it was demonstrated in the UPSTREAM study that this would not change the number of patients undergoing surgical treatment [21]. Another limitation is that the study is unblinded, making detection and performance bias hard to exclude. Due to the physical component of the surgical procedures, blinding of the patients and surgeons has not been possible in our setting. Moreover, the power calculation of this trial is limited by the diverse inclusion criteria of the previous research on which the calculations are based. This could result in a larger SD than assumed, making it infeasible to show non-inferiority with the calculated sample size. Another limitation is that we only include patients with severe LUTS, as less symptomatic patients may have less pronounced improvement. However, contemporary publications have stated the need for subgroup analyses to detect patients who benefit most from MIST [7]. Also, the patients who would receive these interventions as part of their usual care would mainly fall into the group of severe LUTS. We have therefore decided to include only this subgroup of patients to be able to extrapolate our results to the clinic. We hope to build future trials including other subgroups of patients with moderate and mild LUTS.

With this pragmatic head-to-head comparative trial, including long-term follow-up, we aspire to clarify the effectiveness of TUMT and PAE in treating BOO. We expect non-inferiority in the two treatment arms. With analysis of our secondary outcomes, we hope to guide clinicians in patient counselling and selection when choosing the optimal treatment for this condition.

## Trial status

Protocol version 3.0 was approved on December 22, 2022. Recruitment for the trial began in October 2022; we enrolled the first patient on October 27, 2022. The enrolment is expected to be completed in December 2025. The trial in its entirety is expected to run for 8 years, completing the study by December 2030.

## Data Availability

Access to the final trial dataset can be arranged upon request of the CI or person in charge of the trial. Approval from the Danish Data Protection Agency is required to receive data.

## Abbreviations

AE: Adverse event
BII: Benign Prostatic Hyperplasia Impact Index
BOO: Bladder outlet obstruction
BPH: Benign prostatic hyperplasia
CI: Coordinating investigator
CTA: Computed tomography angiography
DAG: Data access group
DAN-PSS-1-sex: Danish Prostatic Symptom Score—sexual questions
DAP: Dose-area product
ED: Erectile dysfunction
GDPR: General Data Protection Regulation
Gy: Gray
ICH GCP: International Council for Harmonisation of Technical Requirements for Pharmaceuticals for Human Use - Good Clinical Practice
ICIQ-UI-SF: International Consultation on Incontinence Questionnaire—Urinary Incontinence Short Form
IIEF: International Index of Erectile Function
IPSS: International Prostate Symptom Score
LUTS: Lower urinary tract symptoms
MIST: Minimally invasive surgical therapy
MRI: Magnetic resonance imaging
mSv: Millisievert
PAE: Prostatic artery embolisation
PES: Post-embolisation syndrome
PI: Principal investigator
PRO: Patient-reported outcome
PROM: Patient-reported outcome measure
PSA: Prostate-specific antigen
PV: Prostate volume
QoL: Quality of life
Qmax: Maximum flow rate
REDCap: Research Electronic Data Capture
SAE: Serious adverse event
SD: Standart deviation
TRUS: Transrectal ultrasound
TUMT: Transurethral microwave thermotherapy
TURP: Transurethral resection of the prostate
